# Stable Patients With STEMI Rarely Require Intensive-Care-Level Therapy After Primary PCI

**DOI:** 10.1016/j.cjco.2021.12.013

**Published:** 2022-01-11

**Authors:** Andrew Caddell, Daniel Belliveau, Andrew Moeller, Ata ur Rehman Quraishi

**Affiliations:** Cardiology Division, Dalhousie University, Halifax, Nova Scotia, Canada

## Abstract

**Background:**

The disposition of patients presenting with ST-elevation myocardial infarction (STEMI) is commonly the coronary care unit. Recent studies have suggested that low-risk STEMI patients could be managed in a lower-acuity setting immediately after percutaneous coronary intervention (PCI). We sought to determine the frequency of downstream intensive-care therapy used in our “stable” STEMI patients post-PCI.

**Methods:**

A single-centre, retrospective review was completed of consecutive patients who underwent primary PCI for STEMI between 2013 and 2016. Post-PCI, patients were defined as being stable if they had not required intensive-care therapy or suffered significant complications. Intensive-care therapies and complications were defined as invasive/noninvasive ventilation, pacing, cardiac arrest, use of vasopressors/inotropes, dialysis, stroke, or major bleeding. This group of stable patients had their course followed to discharge.

**Results:**

A total of 731 patients presented with STEMI for primary PCI. Of these, 132 patients (18%) required intensive-care therapies and/or had complications prior to PCI and were excluded. After PCI, 599 STEMI patients (82%) were defined as stable, according to the above definition. Of these, 11 patients (1.8%) required intensive-care therapies during their hospitalization. Zwolle scores were significantly higher in patients with complications (6.3 ± 4.4 vs 2.0 ± 1.5, *P* < 0.0001). The most frequent intensive-care complications and therapies were cardiac arrest (7 patients, 1%) and vasopressor use (4 patients, 0.7%). These complications most frequently occurred on the first admission day (6 patients, 1%).

**Conclusions:**

Patients who are stable at the completion of their primary PCI rarely develop complications that require intensive care. These patients are easily identified for triage to a lower-acuity setting, alleviating congestion in cardiac care units and reducing hospitalization costs.

## Graphical abstract

**Figure:**
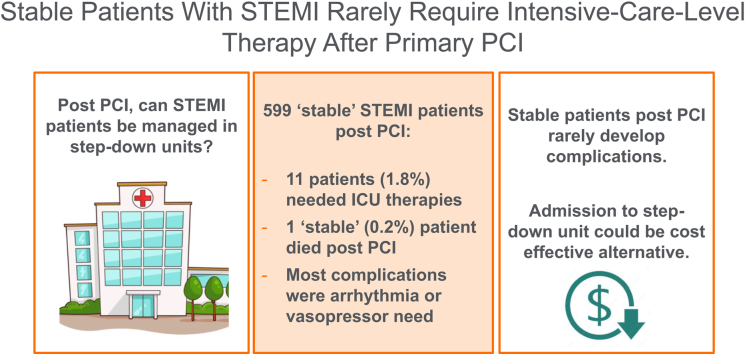

## Résumé

**Introduction:**

Les patients qui subissent un infarctus du myocarde avec élévation du segment ST (STEMI) aboutissent souvent à l’unité de soins coronariens. Des études récentes ont montré que les patients exposés à un faible risque de STEMI pouvaient être pris en charge dans les soins de faible acuité immédiatement après l’intervention coronarienne percutanée (ICP). Nous avons cherché à déterminer la fréquence des traitements de soins intensifs en aval utilisés après notre ICP chez les patients STEMI qui étaient dans un état stable.

**Méthodes:**

Une étude rétrospective unicentrique a été réalisée auprès de patients consécutifs qui subissaient une ICP primaire en raison d’un STEMI entre 2013 et 2016. Après l’ICP, les patients étaient considérés être dans un état stable s’ils n’avaient pas besoin de traitements de soins intensifs ou ne souffraient pas de complications importantes. Les traitements de soins intensifs et les complications étaient définis par la présence de la ventilation effractive ou non effractive, de la stimulation cardiaque, de l’arrêt cardiaque, de l’utilisation de vasopresseurs ou d’inotropes, de la dialyse, de l’accident vasculaire cérébral et de l’hémorragie majeure. Ce groupe de patients dans un état stable obtenaient leur sortie de l’hôpital.

**Résultats:**

Un total de 731 patients STEMI ont subi une ICP primaire. Parmi eux, 132 patients (18 %) ont eu besoin de traitements de soins intensifs et/ou ont eu des complications avant l’ICP et ont été exclus. Après l’ICP, 599 patients STEMI (82 %) ont été considérés comme étant dans un état stable, conformément à la définition ci-dessus. Parmi eux, 11 patients (1,8 %) ont eu besoin de traitements de soins intensifs durant leur hospitalisation. Les indices de Zwolle étaient significativement plus élevés chez les patients qui avaient des complications (6,3 ± 4,4 *vs* 2,0 ± 1,5, *P* < 0,0001). Les complications et les traitements aux soins intensifs les plus fréquents étaient l’arrêt cardiaque (sept patients, 1 %) et l’utilisation de vasopresseurs (quatre patients, 0,7 %). Ces complications sont survenues plus fréquemment à la première journée de l’admission (six patients, 1 %).

**Conclusions:**

Les patients qui sont dans un état stable après l’ICP primaire ont rarement des complications qui exigent des soins intensifs. L’orientation de ces patients, facilement repérés au triage, vers des soins de faible acuité, allège la congestion aux unités de soins intensifs et réduit les coûts d’hospitalisation.

Outcomes for patients with ST-elevation myocardial infarctions (STEMIs) have improved markedly over the past 20 years,^1^, ^2^, ^3^ likely as a result of early revascularization, better medical therapy, and subsequent reduction in STEMI complications.^4^, ^5^ The spectrum in the acuity of patients presenting to the hospital with STEMI is considerable. Despite the considerable variance in patient outcomes, the disposition for patients presenting with STEMI is most commonly either a coronary care unit (CCU) or an intensive care unit (ICU).^6^ The rationale for this approach is largely historical; prior to the revascularization era, the rate of complications was significantly higher.^6^ The most recent European STEMI guidelines have continued to advocate universal admission to a CCU or ICU for all patients, irrespective of clinical stability.^1^ In contrast, the 2004 American College of Cardiology/American Heart Association STEMI guidelines make a Class I recommendation that lower-risk STEMI patients be admitted directly to step-down units (SDUs) after percutaneous coronary intervention (PCI).^7^

More recent studies^8^ and opinion pieces^9^ have suggested that low-risk STEMI patients could be managed in a lower-acuity setting immediately post-PCI. A Canadian study of low-risk non-ST elevation acute coronary syndrome suggested that these patients could be effectively managed in an SDU.^10^ SDUs that manage low-risk STEMI patients require telemetry, skilled nursing, and the ability to provide emergent defibrillation in case of ventricular arrhythmia.^7^ Triaging low-risk STEMI patients similarly has potential to alleviate congestion in chronically overfilled CCUs.^9^ This approach could also reduce the cost of hospitalization for low-risk STEMI patients.^8^ Unfortunately, only a paucity of Canadian data are available to evaluate this possibility.

We postulate that a subset of low-risk STEMI patients can be managed safely in an SDU immediately post–primary PCI. We sought to establish the frequency with which intensive-care therapies are utilized in our STEMI patients. We next evaluated the clinical course of our “stable” or low-risk STEMI patients to establish criteria for a safe admission to an SDU.

## Methods

The Queen Elizabeth II Health Science Centre provides tertiary and quaternary care to adults within the Maritime Provinces, and it is the only centre in Nova Scotia that performs cardiac catheterization. All patients who have primary PCI for STEMI have their baseline demographics and clinical characteristics recorded in a secure database.

### Patient population and data collection

This single-centre, retrospective review included adult patients (age 18 years and older) who underwent primary PCI between 2013 and 2016. Patients were excluded from the primary analysis if they were “unstable,” requiring intensive-care therapies, or had complications requiring critical care (defined below) prior to PCI completion. Demographic information was extracted from the cardiac catheterization database, including age, sex, comorbidities, ischemic time, type of STEMI, and type of intervention performed. Their course in the hospital was reviewed using the institutional electronic medical record. The Zwolle risk score, a validated scoring system for early discharge post-STEMI, was calculated for each patient as well.^11^ The score uses 6 clinical variables (age, type of STEMI, thrombosis in myocardial infarction flow, presence of 3-vessel disease, Killip class, and ischemic time > 4 hours), and a score of ≤ 3 is considered low-risk.

Intensive-care therapies were defined to include the following: intubation and mechanical ventilation, use of noninvasive positive-pressure ventilation, temporary transvenous or transcutaneous pacing, cardiac arrest or requirement for advanced cardiac life support therapy, use of vasopressors or inotropes, and new use of renal replacement therapy. Critical care complications included stroke and major bleeding from thrombosis in myocardial infarction.

Any patient that did not require intensive-care therapy and did not have a complication by the conclusion of their PCI in the cardiac catheterization suite were defined as being stable. These patients had their clinical course followed until discharge.

This study was approved by the Nova Scotia Health Research Ethics Board.

### Statistical analysis

Continuous variables were expressed as means with interquartile ranges, and categorical variables were expressed as percentages. To compare categorical variables, χ^2^ analysis was used. To compare means, *t* tests were used.

## Results

Between 2013 and 2016, a total of 731 patients presented with STEMI for primary PCI. Of these, 132 patients (18%) required intensive-care-level therapies prior to completion of their cardiac catheterization and were excluded from the stable patient group. For the unstable group, the most frequent intensive-care therapies and complications included cardiac arrest (95 patients, 71%), inotrope and vasopressor use (74 patients, 56%), cardiogenic shock (62 patients, 47%), and intubation (53 patients, 40%). A total of 63% of patients had more than one indication for intensive-care therapy. Demographic information for stable and unstable patients is presented in Table 1.

**Table 1:** Baseline characteristics of “stable” vs “unstable” patients arriving for primary percutaneous coronary intervention

Characteristic	Stable patients (n = 599)	Unstable patients (n = 132)
Age, y	61 ± 12	60 ± 12
Sex, female	23	10
Hypertension	51	52
Diabetes	24	22
Dyslipidemia	50	44
Prior CABG	1	5
Chronic kidney disease	5	8
Anterior MI	39	48
Inferior MI	47	28
Lateral MI	14	24
Ischemic time, min	666 ± 436	661 ± 437
LVEDP, mm Hg	15 ± 11	18 ± 13
LVEF, %	48 ± 9.2	44 ± 13
Multi-vessel disease	39	54
Stents inserted	1.3 ± 0.57	1.3 ± 0.70
Zwolle score	2.0 ± 1.5	6.3 ± 4.4
Intubation	0	40
Cardiogenic shock	0	47
Vasopressors/ inotropes	0	56
ACLS/ cardiac arrest	0	71
NIPPV	0	3
IABP	0	10
VA-ECMO	0	3
Percutaneous LVAD	0	1

Values are mean ± standard deviation, or %. LVEF is based on echocardiography. Multi-vessel disease is critical disease in more than 1 vascular territory.

ACLS, advanced cardiac life support; CABG, coronary artery bypass graft; IABP, intra-aortic balloon pump; LVAD, left ventricular assist device; LVEDP, left ventricular end diastolic pressure; LVEF, left ventricular ejection fraction; MI, myocardial infarction; NIPPV, noninvasive positive-pressure ventilation; VA-ECMO, veno-arterial extracorporeal membrane oxygenation.

Stable patients had significantly lower in-hospital mortality (0.17% vs 14%, *P* < 0.0001) and a shorter length of stay (4 ± 2.5 days vs 9 ± 19 days, *P* < 0.0001), compared with patients who presented as unstable.

**Figure 1:**
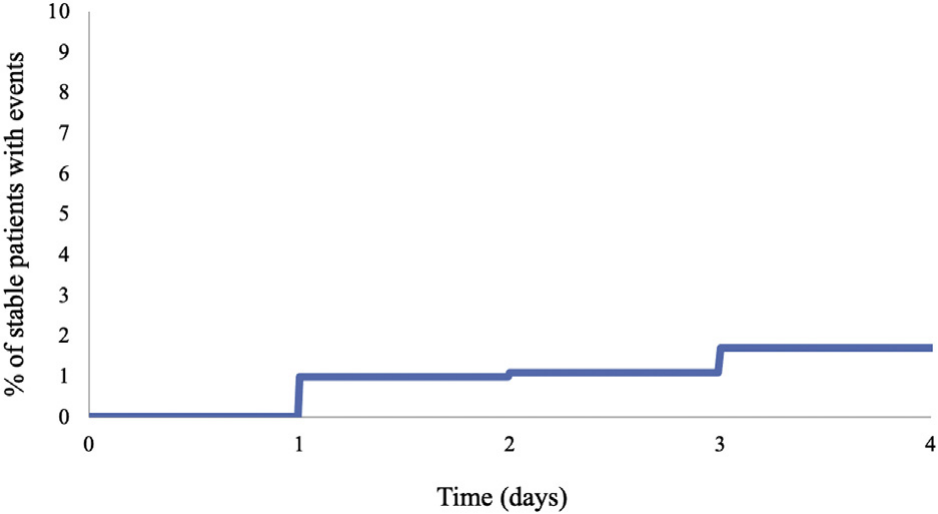
Time to requirement of intensive-care therapies or complications (days) for “stable” ST-elevation myocardial infarction patients post–percutanous coronary intervention.

At the conclusion of primary PCI, 599 patients (82%) were defined as stable. In this group, only 11 patients (1.8%) went on to require intensive-care therapies or had complications requiring critical care during their hospitalization. Cardiac arrest occurred in 7 patients (1%), and vasopressors were used in 4 patients (0.7%; Table 2). Intensive-care complications occurred most frequently on the first admission day (6 patients, 1%; Fig. 1). Detailed descriptions of all complications are listed in Appendix 1. Among stable STEMI patients, only one patient died after admission. This patient was a frail 90-year-old whose limited goals of care were clarified after her PCI. Only 1 patient required readmission to a CCU after a ventricular fibrillation/ventricular tachycardia arrest on the SDU on post-admission day 3 (Appendix 1).

**Table 2:** In-hospital outcomes for patients who arrived stable for primary percutaneous coronary intervention

	Stable patients (n = 599)
Cardiogenic shock	0.2 (1)
Intubation	0
NIPPV	0.2 (1)
Temporary pacing	0.2 (1)
Cardiac arrest/ACLS	1 (7)
Vasopressors or inotropes	0.7 (4)
Renal replacement therapy	0
Stroke	0.3 (2)
TIMI major bleeding	0.2 (1)
No intensive care therapy or support	98 (588)

Values are % (n).

ACLS, advanced cardiac life support; NIPPV, noninvasive positive-pressure ventilation; TIMI, thrombosis in myocardial infarction.

Table 3 compares the characteristics of stable STEMI patients who developed complications, with those who did not. In brief, stable patients who went on to develop complications were significantly older, were more likely to have had a coronary artery bypass graft in the past, and had lower ejection fractions. Patients in the stable group who went on to develop complications had a significantly higher Zwolle score than stable patients who were complication-free (6.3 ± 4.4 vs 2.0 ± 1.5, *P* < 0.0001). At the conclusion of PCI, stable patients with a Zwolle score of 3 or less rarely had complications (7 patients, 1%).

**Table 3:** Comparison of “stable” STEMI patients who remained stable vs those who developed complications

Characteristic	Patients without complications (n = 588)	Patients with complications (n = 11)	*P*
Age, y	61 ± 12	69 ± 13	0.03
Sex, female	23	45	0.08
Hypertension	52	45	0.66
Diabetes	24	27	0.80
Dyslipidemia	49	64	0.33
Prior CABG	2	18	0.0004
Anterior MI	29	45	0.25
Ischemic time, min	665 ± 433	755 ± 571	0.51
LVEF, %	48 ± 9	34 ± 14	< 0.0001
Multi-vessel disease	39	64	0.10
Stents inserted	1.3 ± 0.57	1 ± 0	0.08
Zwolle score	2.0 ± 1.5	4.1 ± 2.8	< 0.0001

Values are mean ± standard deviation, or %, unless otherwise indicated.

CABG, coronary artery bypass graft; LVEF, left ventricular ejection fraction; MI, myocardial infarction; STEMI, ST-elevation MI.

## Discussion

In this single-centre, retrospective review of patients who underwent primary PCI for STEMI, we were able to define the frequency of critical care therapies and complications. A minority (18%) of unstable patients accounted for the vast majority of the critical care therapy, morbidity, and mortality for the total group requiring PCI for STEMI. We found an extremely low complication rate in patients who were stable at completion of their PCI. Among almost 600 patients, the downstream use of intensive-care therapies was infrequent, and only a single death occurred. The most frequently used intensive-care therapies in the stable group were advanced cardiac life support for arrhythmia and vasopressor use.

Variability in admission patterns to ICUs and CCUs has been described previously.^5^, ^6^, ^7^, ^8^, ^9^ A small Taiwanese study demonstrated that 58 low-risk STEMI patients were admitted to an SDU with good clinical outcomes and a reduction in healthcare system costs.^8^ A large American registry study failed to demonstrate differences in outcomes between ICU and non-ICU admissions for STEMI.^6^ Opinion pieces have suggested that low-risk STEMIs post–primary PCI patients are generally inappropriate for CCU/ICU admission.^9^ Canadian data has shown that STEMI continues to be the predominant admission diagnosis to CCUs, despite falling mortality rates.^12^

Definitive criteria to determine which STEMI patients are low-risk remain elusive. Risk stratification scores have wide variation in sensitivity, specificity, and predictive values.^13^ Ad hoc physician estimates of benefits to critical care admissions also show poor agreement and often are influenced by nonclinical factors.^14^ This uncertainty of benefit is present in both cardiac and general system ICUs.^14^ Other centres have used greatly simplified metrics, such as ischemic time, but continue to have complication rates as high as 13% in the “low-risk” group.^5^ The Zwolle score potentially can identify low-risk patients for admission to an SDU,^11^ and in our population, a significant difference was found in scores for stable vs unstable patients. Further validation in this patient population is necessary.

We submit our study as a first step for a simplified triage of patient stability post–PCI for STEMI. If a patient was stable during their PCI, then it is extremely unlikely that their later clinical course will require critical-care-level therapy. Our definition of a patient who is stable requires only clinical assessment at the bedside, and it includes those who are free of invasive or noninvasive ventilation, significant hemodynamic or electrical instability, and prior cardiac arrest. In this group, we observed a complication rate of 2%, and a mortality rate of 0.2%. In addition, our stable patient cohort can be further stratified by use of the Zwolle score to identify patients potentially at very low risk of decompensation. Regular vitals and routine telemetry could capture arrhythmogenic and hemodynamic complications in these patients.^1^ Although some centres^15^ and guidelines^1^ have advocated 24 hours of CCU admission for all STEMI patients, our stable STEMI group had a complication rate of only 1% in the first 24 hours. Also noteworthy is that a number of the listed complications were picked up in the SDU with excellent patient outcomes, suggesting that the SDU may be effective in monitoring for STEMI complications. Of the 11 patients with “complications,” 3 actually had their complications managed in an SDU, without requirement for readmission to a CCU or ICU. Subsequent randomized controlled data are needed to ensure that universal CCU admission post-STEMI does not confer an unrecognized survival benefit.

The implications of decanting stable STEMI patients to SDUs across Canada are significant. Decongesting CCUs would allow for reduction in costs, ease strain, and increase critical-care bed availability.^9^ Within CCUs, this approach would afford greater time and resources for patients who would derive benefit from intensive-care-level therapy.^9^ It could also lead to the development of formal appropriateness in admission criteria for acute myocardial infarction, decompensated heart failure, and arrhythmia. Stable patients also could be candidates for early discharge post-PCI, an approach that other Canadian centres have begun trialing in carefully selected patients.^16^

This retrospective study has several limitations. As all patients were admitted to a CCU after their primary PCI, some adverse events may have been avoided as a result of the intensive monitoring and nursing care. There is no local comparator group of patients admitted directly to the SDU. Our patient population did not include pharmacoinvasive or facilitated PCI approaches. We also did not include patients for whom medical therapy for their STEMI was planned, or those who had surgical revascularization. Finally, the definition of stable used for our STEMI group has not been validated in a prospective manner.

## Conclusion

Patients who are stable at the completion of their primary PCI rarely develop complications that require intensive care. These patients are easily identified and could be triaged to a lower-acuity setting, alleviating congestion in CCUs and potentially reducing the cost of hospitalization.

## Supplementary Material

Appendix 1 Detailed description of initially “stable” patients with ST-elevation myocardial infarction (STEMI), with their post-admission complications. CCU, cardiac care unit; GI, gastrointestinal; HFrEF, heart failure with reduced ejection fraction; LAD, left anterior descending artery; LVEF, left ventricular ejection fraction; NIPPV, noninvasive positive-pressure ventilation; PCI, percutaneous coronary intervention; ROSC, return of spontaneous circulation; SDU, step-down unit; TIA, transient ischemic attack; VF, ventricular fibrillation; VT, ventricular tachycardia.
