# Supplementary material for: Stable Patients With STEMI Rarely Require Intensive-Care-Level Therapy After Primary PCI
Source: CJC Open. 2022 Jan 11;4(4):390–4. doi: 10.1016/j.cjco.2021.12.013 (PMC9039553; doi:10.1016/j.cjco.2021.12.013)
Supplement: Appendix 1 — Detailed description of initially “stable” patients with ST-elevation myocardial infarction (STEMI), with their post-admission complications. CCU, cardiac care unit; GI, gastrointestinal; HFrEF, heart failure with reduced ejection fraction; LAD, left anterior descending artery; LVEF, left ventricular ejection fraction; NIPPV, noninvasive positive-pressure ventilation; PCI, percutaneous coronary intervention; ROSC, return of spontaneous circulation; SDU, step-down unit; TIA, transient ischemic attack; VF, ventricular fibrillation; VT, ventricular tachycardia. [file mmc1.pdf]

**Patient 1**	86-year-old woman with an anterior STEMI. 1-hour post-PCI to LAD artery had a VT/VF arrest with ROSC (not intubated). Etiology was stent thrombosis.<br>Discharged home after 7 days in the hospital.
**Patient 2**	70-year-old woman with a lateral STEMI. PCI to circumflex jailed an obtuse marginal. Admitted to CCU for a controlled infarct; had VT/VF 7 hours post-PCI (not intubated).<br>Discharged home after 6 days in the hospital.
**Patient 3**	82-year-old man with inferolateral STEMI. Post-PCI day 5 had an embolic stroke while in the SDU.<br>Discharged home after 20 days in the hospital.
**Patient 4**	67-year-old man with inferior STEMI. Post-PCI day 3 in the SDU had a VT/VF arrest with ROSC after 1 cycle (not intubated). Readmitted to the CCU.<br>Discharged home after 8 days in the hospital.
**Patient 5**	66-year-old man with lateral STEMI. Post-PCI days 3 and 4 he had TIAs without fixed deficits.<br>Discharged home after 6 days in the hospital.
**Patient 6**	61-year-old woman with anterior STEMI. 2 hours post-PCI to LAD artery developed complete heart block in the CCU. Had a temporary transvenous pacemaker inserted, then a permanent one the following day.<br>Discharged home after 3 days in the hospital.
**Patient 7**	69-year-old man with an anteroseptal STEMI. Post-PCI day 3 had VT/VF arrest with ROSC (not intubated) while in the CCU. Prolonged stay for HFrEF medication titrations (LVEF 17%).<br>Discharged home after 17 days in the hospital.
**Patient 8**	46-year-old man with an anterior STEMI. 2 hours post-PCI to his LAD artery, he went into cardiogenic shock requiring NIPPV and inotropes.<br>Discharged home after 13 days in the hospital.
**Patient 9**	59-year-old woman with an anterior STEMI. Post-PCI day 2 she developed an upper GI bleed while on the SDU, requiring 5 units of packed red blood cells.<br>She was discharged home after 14 days in the hospital.
**Patient 10**	65-year-old man with anterolateral STEMI. Post-PCI day 1 had brief VF arrest (not intubated).<br>Discharged home after 8 days in the hospital.
**Patient 11**	90-year-old woman with a lateral STEMI. Frail, from a nursing home, with pre-expressed wishes to avoid hospital-based therapy. Post-PCI had hypotension and early cardiogenic shock. Her goals of care were adjusted in light of her values, and she died on the day of admission.
